# Effect of osteoconductive hyaluronate hydrogels on calvarial bone regeneration

**DOI:** 10.1186/2055-7124-18-8

**Published:** 2014-07-23

**Authors:** Junseok Yeom, Byung Woo Hwang, Dong Jun Yang, Hong-In Shin, Sei Kwang Hahn

**Affiliations:** Department of Materials Science and Engineering, Pohang University of Science and Technology (POSTECH), San 31, Hyoja-dong, Nam-gu, Pohang, Kyungbuk 790-784 Korea; MegaGen Research Institute of Science and Technology, 377-2 Gyochon-ri, Jain-myeon, Kyeongsan, Kyungbuk 712-852 Korea; Department of Oral Pathology, School of Dentistry, IHBR, Kyungpook National University, 188-1, Samdeok-dong, Jung-gu, Daegu, Kyungbuk 700-412 Korea

**Keywords:** Hyaluronic acid, Gelatin, Hydrogel, Synthetic bone, Bone regeneration

## Abstract

**Background:**

Without exploitation of possibly immunogenic and carcinogenic bone morphogenetic protein, we developed simple but clinically feasible artificial bone graft using osteoconductive hyaluronate (HA) hydrogels and bioactive MegaGen synthetic bone (MGSB).

**Methods:**

HA hydrogels were synthesized by the crosslinking reaction between carboxyl groups of HA and amine groups of gelatin (GEL). Then, artificial bone grafts were prepared by mixing MGSB with HA-GEL hydrogels. The bone regeneration by the MGSB/HA-GEL hydrogel complex was assessed in the skull of New Zealand white male rabbits in 4 and 8 weeks.

**Results:**

HA hydrogels were synthesized by the crosslinking reaction between carboxyl groups of HA and amine groups of gelatin (GEL). Then, artificial bone grafts were prepared by mixing MGSB with HA-GEL hydrogels. *In vitro* proliferation of preosteogenic cells was enhanced with increasing molecular weight of HA. In addition, histological analysis of dissected tissues with hematoxylin and eosin staining confirmed the effective *in vivo* bone regeneration by the MGSB/HA-GEL hydrogel complex. The MGSB/HA-GEL hydrogels were well resorbed and partially substituted to the lamellar bone after implantation for 8 weeks.

**Conclusions:**

The novel artificial bone graft of MGSB/HA-GEL hydrogel complex for effective bone regeneration might be clinically feasible for further development.

## Background

The repair of a bone fracture is a spontaneous, proliferative, and physiological process where the woven bone is formed, subsequently replaced to lamellar bone, and finally remodeled into compact bone [1–4]. However, the bone regeneration is not easy for the cases of severe diseases or large orthopedic defects [5, 6]. In order for that, artificial bone grafts can be crucial for quick and stable ossification of the broken tissue [7]. A great variety of attempts have been made to develop bone filler materials for the effective bone regeneration using allografts, synthetic or natural polymers, and bioceramics [8]. In particular, ceramic based bone grafts have been regarded as one of the most successfully commercialized and widely prevalent biomaterials including Bio-Oss® made of the inorganic portion of bovine bone, hydroxyapatite-based bone graft of Pro-Osteon™, synthetic biphasic calcium phosphate of MBCP™, and bioactive Bone Plus™ of MegaGen synthetic bone (MGSB) [2]. Despite the wide applications of bone grafts, poor osteoconduction and slow bioresorption in the initial intramembranous ossification made discrete and precarious boundaries at the newly formed bone tissue [9].

On account of these clinical issues, there have been plenty of attempts to utilize growth factors like recombinant human bone morphogenetic protein (rhBMP), or mesenchymal stem cells (MSC) with bone grafts [10, 11]. However, these methods are complicated, possibly causing safety issues of immune reaction, and expensive for further clinical applications. Instead of these systems, we tried to develop a novel hybrid bone graft consisted with bioactive calcium phosphate synthetic bone of MGSB and natural biopolymers of hyaluronate (HA) and gelatine (GEL). HA is a biodegradable, biocompatible, non-immunogenic, and natural linear polysaccharide in the body. In addition, HA is known to be angiogenic and osteoconductive, contributing to the effective bone tissue regeration [11–13]. According to the most recent reports, it was demonstrated that MSCs are migrated, granulated, and differentiated to the osteoblastic cells in the presence of the low molecular weight HA [14]. In addition, small fragments of HA with several repeating units can promote angiogenesis significantly [15, 16], which is necessary for replacing the fragile woven bone to the sustainable lamellar bone [4].

In this work, we prepared a novel bone graft complex of MGSB and HA-GEL hydrogel. HA-GEL hydrogels were synthesized and compared with HA-divinyl sulfone (HA-DVS) hydrogels in terms of degradation kinetics. HA hydrogels were thought to continuously provide low molecular weight HA fragments by the degradation in the body, serving as a perfect way to facilitate and advance the early ossification and the sequential bone regeneration. The hydrolysed collagen of GEL was used as a crosslinker due to the hemostatic properties [17]. Meanwhile, the effect of the molecular weight of HA was investigated on the proliferation of preosteogenic cells of MC3T3 on the bioactive MGSB. After implantation of four different samples of a control, MGSB, MGSB/HA, and MGSB/HA-GEL hydrogel to the calvarial critical sized bone defects in the skull of New Zealand white male rabbits, the bone regeneration was assessed by histological analysis with hematoxylin and eosin (H&E) staining in 4 and 8 weeks.

## Methods

### Materials

Sodium hyaluronate (HA), sodium salt of hyaluronic acid (234 kDa), was purchased from Lifecore (Chaska, MN). Gelatin (GEL) was purchased from Yakuri Pure Chemicals(Kyoto, Japan). Phosphate buffered saline (PBS) tablet, H&E, glutathione, and hyaluronidase from Streptomyces hyalurolyticus were purchased from Sigma-Aldrich (St. Louis, MO). 1-Ethyl-3-[3-(dimethylamino)propyl] carbodiimide (EDC), divinyl sulfone (DVS) were purchased from Tokyo Chemical Industry (Tokyo, Japan). Hydrochloric acid and sodium hydroxide were obtained from Wako Pure Chemical Industries (Osaka, Japan). All the chemicals were used without further purification.

### Synthesis of hydrogels

HA-DVS hydrogels were synthesized as we previously reported elsewhere [18]. HA (100 mg) was dissolved in 0.2 N sodium hydroxide (2.5 ml) and DVS (26.47 μl) was added to the HA solution. After the Michael addition reaction between hydroxyl groups of HA and double bonds of DVS for 1 h, the HA-DVS hydrogels were sealed within prewashed dialysis membrane (MWCO of 7 kDa) and dialyzed against PBS for 24. HA-GEL hydrogels were prepared by carbodiimide reaction between carboxyl groups of HA and amine groups of GEL. HA (100 mg) and GEL (72.5 mg) were dissolved in DI water (1.25 ml), respectively, and the two solutions were mixed for 6 h. Then, EDC (191.7 mg) and sulfo-NHS (54.1 mg) were added for the crosslinking reaction overnight. The HA-GEL hydrogels were sealed within prewashed dialysis membrane (MWCO of 7 kDa) and dialyzed against PBS for 72 h.

### *In vitro*hydrogel degradation test

Two kinds of HA hydrogel samples described above were prepared in syringes for *in vitro* degradation tests. Each of HA-DVS hydrogel and HA-GEL hydrogel was put into a vial, respectively. Then, sodium phosphate buffer (0.2 M, pH = 6.2) containing 40 U of hyaluronidase was added to the vials. The samples were incubated at 37°C for the predetermined times (0–36 h). After that, the supernatant was completely removed and the remaining weight of HA hydrogels was measured with a balance. The degree of HA hydrogel degradation was represented by the weight ratio (%) of the remaining hydrogel to the original hydrogel. Triplicates were carried out for each sample.

### *In vitro*cell proliferation assay

MC3T3-E1 cells were seeded into 24 well cell culture plate at an initial density of 5 × 10^3^ cells per well with 200 mg of artificial bone graft (MGSB), which were incubated at 37°C in cell culture media (α-MEM, 10% FBS, 1× antibiotics). After incubation for 7 days, the spherical bone grafts were fixed with 4% paraformaldehyde and washed several times, and serially dehydrated to 100% ethanol. Then, the cells on MGSBs were observed by scanning electron microscopy (SEM, Philips electron optics) after gold coating. The proliferation of MC3T3 cells was assessed by MTT assay at the predetermined times (3, 5, and 7 days).

### *In vivo*implantation and histological analysis

Three New Zealand white male rabbits weighing about 4 kg were used per each experimental group. They were anesthetized by intramuscular injection of zoletil and rompun (v/v = 1/1, 0.1 cc/kg). Two critical sized bone defects of each New Zealand White male rabbit with a diameter of 9 mm were made as we described elsewhere [18]. HA-GEL hydrogels were completely homogenized to micro-sized hydrogels with a homogenizer (T-18 basic; IKA, Tokyo, Japan) at 8000 rpm for 5 min and mixed with MGSB (40 mg of MGSB and 100 μl of microhydrogel), which were inserted into the calvarial critical-sized bone defects. For comparison, the bone defects were also filled with MGSB, MGSB/HA, or remained without graft as a nongrafted control. The rabbits were sacrificed for histological and histomorphometric analyses after H&E staining (n = 3 for each sample) in 4 and 8 weeks. The regenerated bone defect samples were fixed with 10% formalin for 2 days and decalcified with 10% ethylenediaminetetraacetic acid for 2–3 weeks. The degree of bone regeneration was assessed by observation with a digital camera-connected light microscope (Olympus, Tokyo, Japan). We complied with the POSTECH institutional ethical protocols for animals.

## Results and discussion

**Figure 1:**
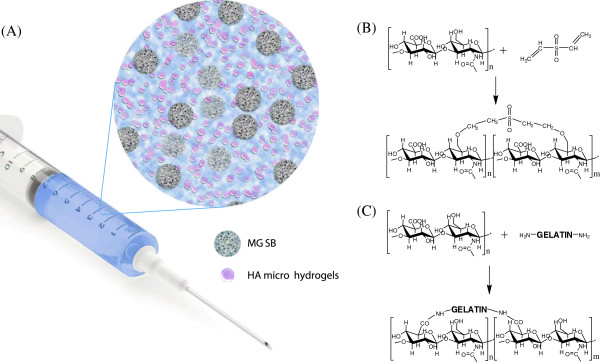
**Schematic representation of (A) artificial bone graft composed of bioactive MGSB and HA microhydrogels, and the synthesis of (B) HA-DVS and (C) HA-GEL hydrogels.**

To take advantages of osteoconductive HA, we developed an artificial hybrid bone graft of MGSB and HA-GEL hydrogels for bone tissue engineering applications. HA hydrogels were designed to provide HA fragments continuously for effective bone regeneration. As schematically shown in Figure 1, we prepared HA microhydrogels mixed with synthetic bone graft of MGSB. Two types of HA hydrogels were prepared by the crosslinking with DVS and GEL for comparison. HA-DVS hydrogels were synthesized by the Michael addition reaction between double bond of DVS and hydroxyl group of HA in a basic solution (Figure 1B). HA-GEL hydrogels were synthesized by simple EDC chemistry between carboxyl groups of HA and amine groups of GEL (Figure 1C).

**Figure 2:**
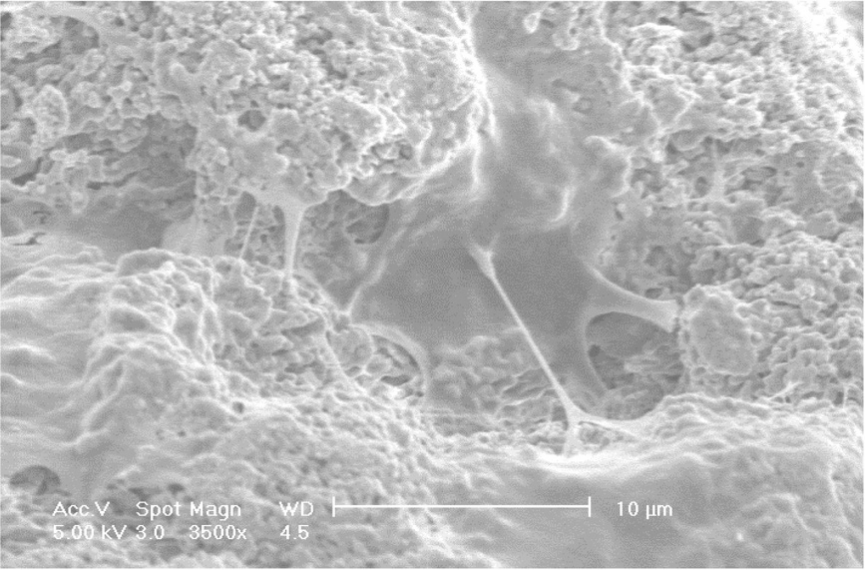
**Scanning electron microscopic image of MC3T3-E1 cells proliferated on the surface of MGSB after gold coating.**

**Figure 3:**
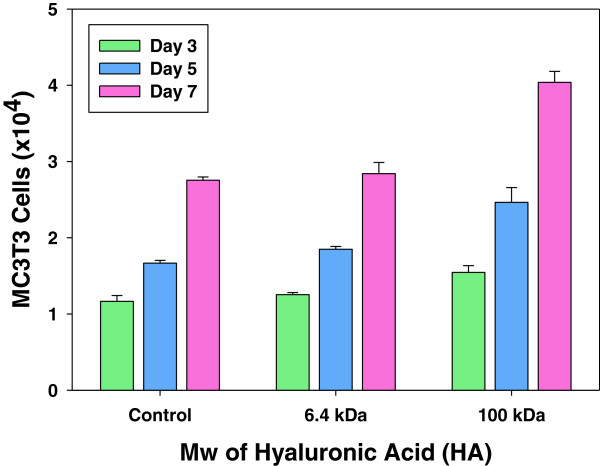
**Effect of the molecular weight of HA on the proliferation of MC3T3-E1 cells.**

Figure 2 shows the SEM image of MC3T3-E1 cells cultured on the artificial bone graft of MGSB, reflecting the biocompatibility of MGSB. The preosteoblast was well attached and proliferated to the surface of microporous bone grafts (Figure 2). Then, we investigated the effect of HA on the proliferation of MC3T3-E1 cells on MGSB. Interestingly, the cell proliferation was higher in the presence of HA with a molecular weight of 100 kDa than 6.4 kDa (Figure 3). The results were well matched with other reports on the effect of HA molecular weight on *in vitro* cell viability and cell proliferation [11, 19]. The more effective bone regeneration in the case of high molecular weight HA might be ascribed to the relatively long-term delivery of the small fragments of HA, corroborating the rationale of the artificial hybrid bone graft of MGSB and HA hydrogels.

**Figure 4:**
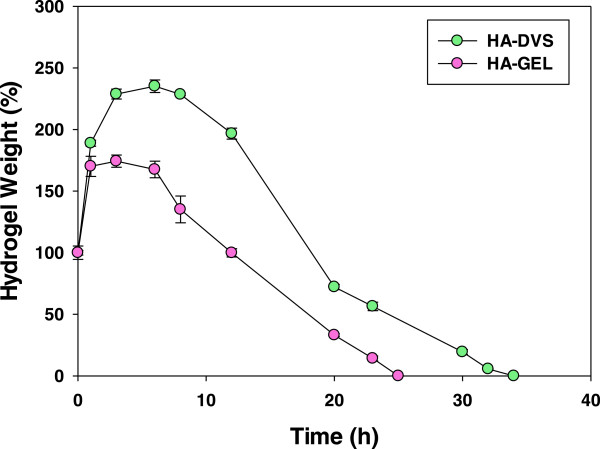
***In vitro*** **degradation of HA-DVS and HA-GEL hydrogels in the presence of hyaluronidase.**

Low molecular weight HA is known to play an important role in bone regeneration, promoting the differentiation [11, 20], vascularization [15, 16], and migration [4, 14] of MSCs. In this respect, we designed a fast degradable HA hydrogel to supply small fragment of HA for the early time of bone regeneration. We compared the degradation of HA-GEL hydrogel with HA-DVS hydrogel after treatment with hyaluronidase. HA-GEL hydrogels with a lower crosslinking density were degraded faster than HA-DVS hydrogels (Figure 4). In addition, GEL might be more vulnerable than DVS during the hyaluronidase treatment [21]. Especially, the degradation of HA-GEL hydrogel can be enhanced in the body due to the GEL degrading enzymes such as matrix metalloproteases (MMP). HA-DVS hydrogels with remaining carboxyl groups swelled more than HA-GEL hydrogels [21]. The relatively slow degradation and high swelling of HA-DVS hydrogels might not be advantageous for bone regeneration, inhibiting the attachment and proliferation of osteoblast cells *in vivo* [18].

**Figure 5:**
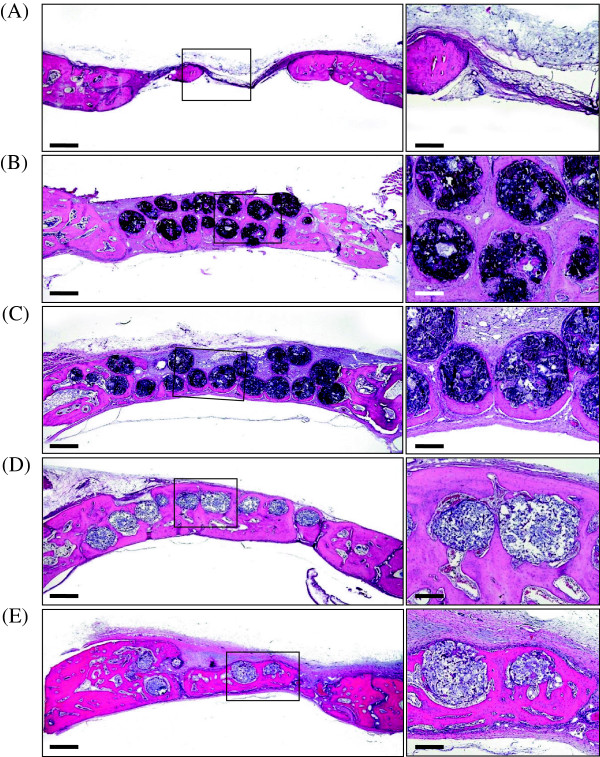
**Photomicrographs if the calvarial critical-sized bone defects in New Zealand white rabbits after bone regeneration for 4 weeks: (A)** **No treatment,** **(B)** **MGSB only,** **(C)** **MGSB/HA, and** **(D)** **MGSB/HA-GEL hydrogel.** **(E)** MGSB/HA-GEL hydrogel after bone regeneration for 8 weeks. Scale bars: left, 1000 μm; right, 200 μm.

The calvarial critical sized bone defects were formed in the New Zealand white rabbits to assess the effect of PBS, MGSB only, MGSB/HA solution, and MGSB/HA-GEL hydrogel on the bone regeneration (Figure 5). We could confirm the effective bone regeneration by the osteoconductive HA (Figure 5B and C). The regenerated bone by MGSB/HA was well matured around the MGSB, whereas that by MGSB only was partially segregated. Figure 5D shows the more effective bone regeneration by MGSB/HA-GEL hydrogels than those by the control samples. The newly formed bone was well interconnected to the MGSB after bone regeneration for 4 weeks. Remarkably, as shown in Figure 5E, MGSB was partially degraded and substituted to the lamellar bone structure after bone regeneration for 8 weeks. It was thought that osteoblast and MSC were recruited and proliferated at the initial bone regeneration, followed by the sequential replacement with the lamellar bone.

More than half century, various bone grafts such as hydroxyapatite [10], tricalcium phosphate [10], bioactive glass [22], and poly(methylmethacrylate) [23] have been developed for bone tissue engineering applications. Despite the wide clinical applications, these bone grafts were not sufficient for the effective bone regeneration. Conclusively, HA-GEL hydrogels mixed with MGSB might improve the formation of the initial callus by providing osteoconductive HA fragments with increasing degradation in the body for the effective bone regeneration. HA and GEL have been widely used as a bone scaffold enhancing cell proliferation and modulating bone differentiation [11]. Although BMPs enable accelerated bone regeneration, this approach is not clinically feasible because they are immunogenic and cause a cancer in some cases. In contrast, the simple hybrid bone graft of MGSB and HA-GEL hydrogels resulted in effective bone regeneration, reflecting the feasibility for further clinical applications.

## Conclusions

We successfully developed an artificial bone graft composed of MGSB and HA hydrogels crosslinked with gelatin. The HA-GEL hydrogel was prepared to supply HA continuously during the bone regeneration. The MGSB/HA-GEL hydrogels resulted in the effective bone regeneration after implantation to the critical-sized calvarial bone defect in the skull of New Zealand white rabbits for 4 weeks. The MGSB in the complex was remodeled to the new bone by the homeostasis of mature bone, namely osteogenesis and osteoclasis in 8 weeks. The novel MGSB/HA-GEL hydrogel complex might be feasible for further clinical applications.

## Availability of supporting data

There was no available supporting data.
